# Risk of cancer in acromegaly patients: An updated meta-analysis and systematic review

**DOI:** 10.1371/journal.pone.0285335

**Published:** 2023-11-30

**Authors:** Zhehao Xiao, Pingping Xiao, Yong Wang, Chen Fang, Yong Li

**Affiliations:** 1 Department of medical oncology, The First Affiliated Hospital of Nanchang University, Nanchang, China; 2 College of Physics Science and Engineering Technology, Yichun University, Yichun, China; CNR, ITALY

## Abstract

The incidence of cancer in acromegaly patients may be higher than that in the general population, although this has not been fully elucidated yet. This study analyzed the risk of various important types of cancer in acromegaly patients. The study was registered in INPLASY (registration number: INPLASY202340037). The PubMed, Web of Science, and EMBASE databases were searched for studies based on strict inclusion and exclusion criteria, from the time of database inception up to June 30, 2022. All observational studies of acromegaly patients with cancer were included, without language restrictions. We used the Newcastle–Ottawa scale (NOS) checklist to assess the quality of evidence. A meta-analysis revealed the relationship between acromegaly and cancer using the standardized incidence rates (SIRs) and 95% confidence intervals (CIs) retrieved from the included studies. Nineteen studies were included and analyzed. The overall incidence of cancer (SIR = 1.45, 95%CI = 1.20–1.75), as well as that of thyroid (SIR = 6.96, 95%CI = 2.51–19.33), colorectal and anal (SIR = 1.95, 95%CI = 1.32–2.87), brain and central nervous system (SIR = 6.14, 95%CI = 2.73–13.84), gastric (SIR = 3.09, 95%CI = 1.47–6.50), urinary (SIR = 2.66, 95%CI = 1.88–3.76), hematological (SIR = 1.89, 95%CI = 1.17–3.06), pancreatic and small intestine (SIR = 2.59, 95%CI = 1.58–4.24), and connective tissue (SIR = 3.15, 95%CI = 1.18–8.36) cancers, was higher among patients with acromegaly than among the general population. No association between acromegaly and hepatobiliary, respiratory, reproductive, skin, breast, or prostate cancer was observed. This study demonstrated that acromegaly patients have a modestly increased chance of cancer as compared to the general population. Risk factors for cancer need to be further explored to monitor patients with acromegaly at a high risk for cancer more carefully.

## Introduction

Acromegaly is a rare endocrine disease characterized by excessive release of growth hormone (GH) and insulin-like growth factor 1 (IGF-1), mainly due to GH-secreting pituitary adenomas. Patients usually present with bone, joint, skin, and soft tissue changes that can cause systemic multisystem and tumor-related complications [1]. Conventionally, acromegaly patients may have a higher mortality rate than the general population. With improvements in treatment, the survival rate of patients with acromegaly has increased. However, with the prolonged life expectancy of the aforementioned population, chronic multisystem damage and malignant tumor-related complications have become the leading causes of mortality [2].

Experimental studies have revealed that GH–IGF-1 signaling plays a significant role in the growth of cancer in patients with acromegaly, by affecting cell proliferation. Nevertheless, whether cancer rates are increased due to acromegaly remains controversial in clinical studies [3]. A number of studies have indicated that patients with acromegaly have an increased incidence of cancer, particularly thyroid and colorectal cancers [4, 5]. A few studies have reported a high incidence of breast cancer [6]. However, some studies have shown that the incidence of cancer in patients with acromegaly is similar to or even lower than that in the local population. A large meta-analysis [7] has shown an increased rate of cancers, mainly thyroid and colorectal cancers, in acromegaly patients. However, the effects of other specific cancer categories and sex on cancer incidence remain inconclusive.

We herein conducted a meta-analysis of the standardized incidence rates (SIRs) and 95% confidence intervals (CIs) of cancers overall and of various significant cancers related to acromegaly, to evaluate the risk and tendency for cancer in patients with this disease, qualitatively. This study was registered in INPLASY (registration number INPLASY202340037) (S1 File).

## Materials and methods

### Search strategy and data extraction

Relevant studies published from the time of database inception up to June 30, 2022, were obtained from the PubMed, Web of Science, and EMBASE databases. We combined the following text words and MeSH terms as the search strategy: (“Neoplasm*” OR “Tumor*” OR “Neoplasia*” OR “Cancer*” OR “carcinoma*” OR “malignancy*” OR “tumor*”) AND (“Acromegaly” OR (“Somatotropin Hypersecretion Syndrome*”).

We included studies that reported SIRs and 95%CIs, that included patients diagnosed with acromegaly, and in which the cancer incidence rate of representative populations was used to calculate the expected number of SIRs. We excluded studies that were not original articles, such as case reports and reviews, that involved non-representative populations, including patients with specific diseases, for calculating the expected number of SIRs.

Two researchers searched the aforementioned databases independently and recorded data on the author, publication year and country, data origin, calendar period, number of acromegaly and cancer patients, and follow-up years. We evaluated the quality of articles using the Newcastle–Ottawa scale (NOS) checklist, and studies that scored ≥ 6 were considered to have high quality [8]. The degree of agreement between the researchers was evaluated by calculating Cohen’s kappa. In cases of differences in the selection and data extraction of articles, the two researchers would discuss the issue at hand until a consensus was reached.

### Statistical analysis

SIRs, including 95%CIs, were used to evaluate the association between acromegaly and cancer. The I^2^ test was used to assess heterogeneity. When heterogeneity was notable (*P* < 0.1, I^2^ > 50%), a random-effects model was used. Otherwise, a fixed-effects model was used [9]. Publication bias was weighed using Egger’s test, and a *P*-value < 0.05 was considered to indicate publication bias. If publication bias existed, a trim-and-fill analysis was used to evaluate the effect of publication bias on the pooled SIRs and 95%CIs. One article was excluded each time, and a meta-analysis pooled the remaining articles (n-1 articles) to assess the robustness and reliability of the pooled results. All tests were two-sided, and *P*-value < 0.05 was regarded as statistically significant [10]. All statistical analyses were performed using STATA version 17.0 (Stata Corp, College Station, TX, USA).

## Results

### Description of studies in our meta-analysis

**Fig 1:**
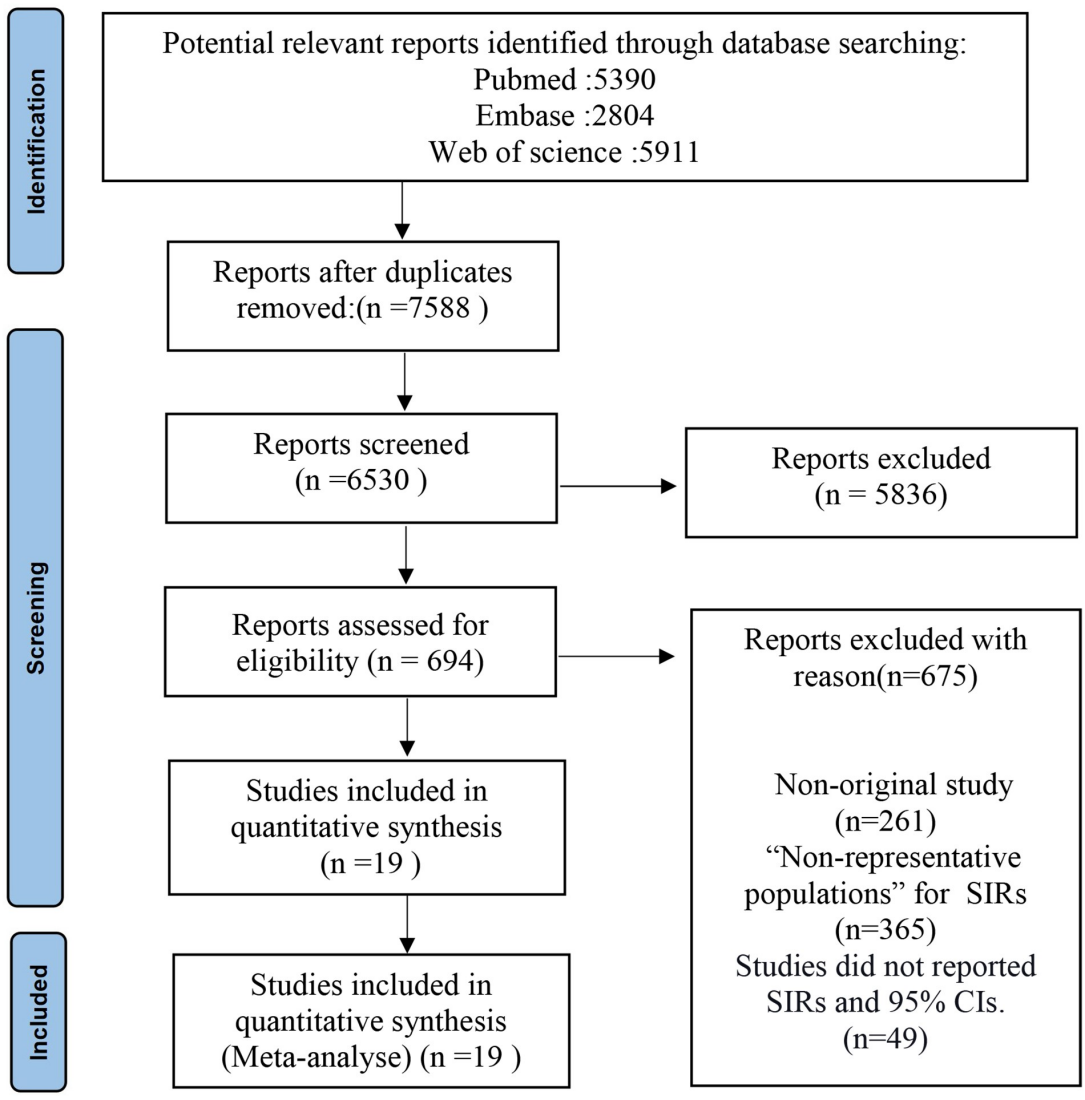
Study selection flowchart.

A total of 14,105 articles were retrieved from the three databases, and 7,588 remained after removing duplicate articles. By reading the title and abstract, we removed 5,836 articles because they were unrelated to acromegaly and cancer. We then read the remaining articles in full. Based on strict inclusion and exclusion criteria, we excluded studies that did not include “representative populations” or that were unable to provide SIRs and 95%Cis (Fig 1).

### Characteristics of studies and patients

Nineteen eligible studies, comprising 11,494 patients, from the inception of these databases to 2022, were enrolled. The study with the highest sample size included 1,634 patients and was a population-based survey from Denmark and Sweden published by Baris et al. [11] in 2002. A multi-center survey conducted in Japan by Huiguchi et al. [12] in 2000 had the smallest sample size (40 individuals). Most studies were conducted in Europe and Asia. Six were single-center studies, eight were multi-center, and five were population-based. Fifteen studies reported their follow-up time, and only 4 studies did not mention follow-up duration. All studies received a score of 6 or higher on the Newcastle–Ottawa scale, meaning that all studies were of high quality. Cohen’s kappa for all evaluation items were higher than 0.61, among which that for 5 items were higher than 0.81, indicating strong inter-researcher consistency (S1 Table).

### Organization of cancer groups

A total of 1,108 acromegaly patients were diagnosed with cancer. Most cancer diagnoses were based on national databases, cancer registries, or pathological diagnoses. A small number of diagnoses were based on the patients’ claims. Only 2 studies [7, 11] excluded patients diagnosed with cancer during the first year after acromegaly diagnosis. Another study [13] included all patients diagnosed with cancer at least 4 years after acromegaly diagnosis. Five studies [14–18] excluded patients who developed cancer before acromegaly diagnosis. Three studies excluded patients whose cancers were diagnosed 6 [5], 5 [19], and 1 [20] year before the diagnosis of acromegaly. One study [21] excluded patients who died in the first year after acromegaly diagnosis, three studies did not exclude patients diagnosed with cancer before acromegaly diagnosis, and four studies did not state whether such patients were included (Table 1).

**Table 1:** Characteristics of studies included in the meta-analysis.

First author	Year	Country	Data origin	Calendar period	Number of ACRO patients (female%)	Number of cancer patients	Follow–up(years)	NOS scores
Durmuş [14]	2022	Turkey	Anonymized hospital	2008–2019	179 (59)	24	5 ± 3.9	8
Daniela [5]	2021	Sweden	Swedish National Patient Registry	1987–2017	1,296 (52)	186	11.7	8
BekirUcan [22]	2020	Turkey	Diskapi and Numune Training and Research Hospital	2010–2019	280 (57)	19	NA	7
Julien [23]	2020	France	Single center	2000–2015	221 (60)	17	7.35 ± 4.6	6
Wu [15]	2019	China	The National Health Insurance Research Database of Taiwan	1997–2013	1,195 (50)	87	7.28	8
Dal [7]	2018	Denmark	Danish National Patient Registry	1978–2010	529 (49)	81	13.6 ± 8.3	8
Maione [24]	2017	France	The French Registry	1999–2012	999 (54)	102	6.7	7
Terzolo [19]	2017	Italy	Italian nationwide multi–center cohort	1980–2002	1,512 (59)	124	10	7
Petroff [25]	2015	Germany	German Acromegaly Registry	2003–2010	445 (56)	42	15	7
Ritva [21]	2010	Finland	5 university hospitals in Finland	1980–2006	331	48	14.6	7
Makiko [26]	2008	Japan	Tokyo Women’s Medical University Hospital	before 2002	140 (61)	22	NA	7
Baris [11]	2002	Sweden and Denmark	Nationwide registry–based cohorts of patients hospitalized	Denmark 1977–1993; Sweden 1965–1993	1,634 (54)	117	Sweden 10.3; Denmark 9.0	8
Huiguchi [12]	2000	Japan	Chiba University Hospital and tertiary referral hospitals	1976–1998	44 (42)	5	NA	6
Orme [16]	1998	England	15 tertiary referral centers	1958–1995	1,239	79	NA	8
Popovic [27]	1998	Serbia	Tokyo Women’s Medical University	1992–1998	220 (62)	23	4.5 ± 0.4	7
Cheung [20]	1997	Australia	Westmead Hospital	1979–1995	50 (42)	7	8.7	6
Barzilay [13]	1991	America	Lahey Clinic	1957–1988	87 (49)	7	13	6
Brunner [17]	1990	America	Southeastern Michigan and northern Ohio	1935–1985	52 (46)	2	12.5 ± 9	6
Ron [18]	1990	America	All VA hospitals in USA	1969–1985	1,041 (0)	116	8.3	8

ACRO, acromegaly; NOS, Newcastle–Ottawa scale; and VA, Veterans’ Health Administration

### Association between acromegaly and cancers

We independently analyzed the risk of major cancer types, such as thyroid, colorectal and anal, gastric, hepatobiliary, pancreas and small intestine, prostate, and breast cancers in patients with acromegaly. For other types of cancer, we classified each cancer into brain and central nervous system, urinary system, reproductive system, hematologic system, respiratory system, connective tissue, and skin cancer.

**Fig 2:**
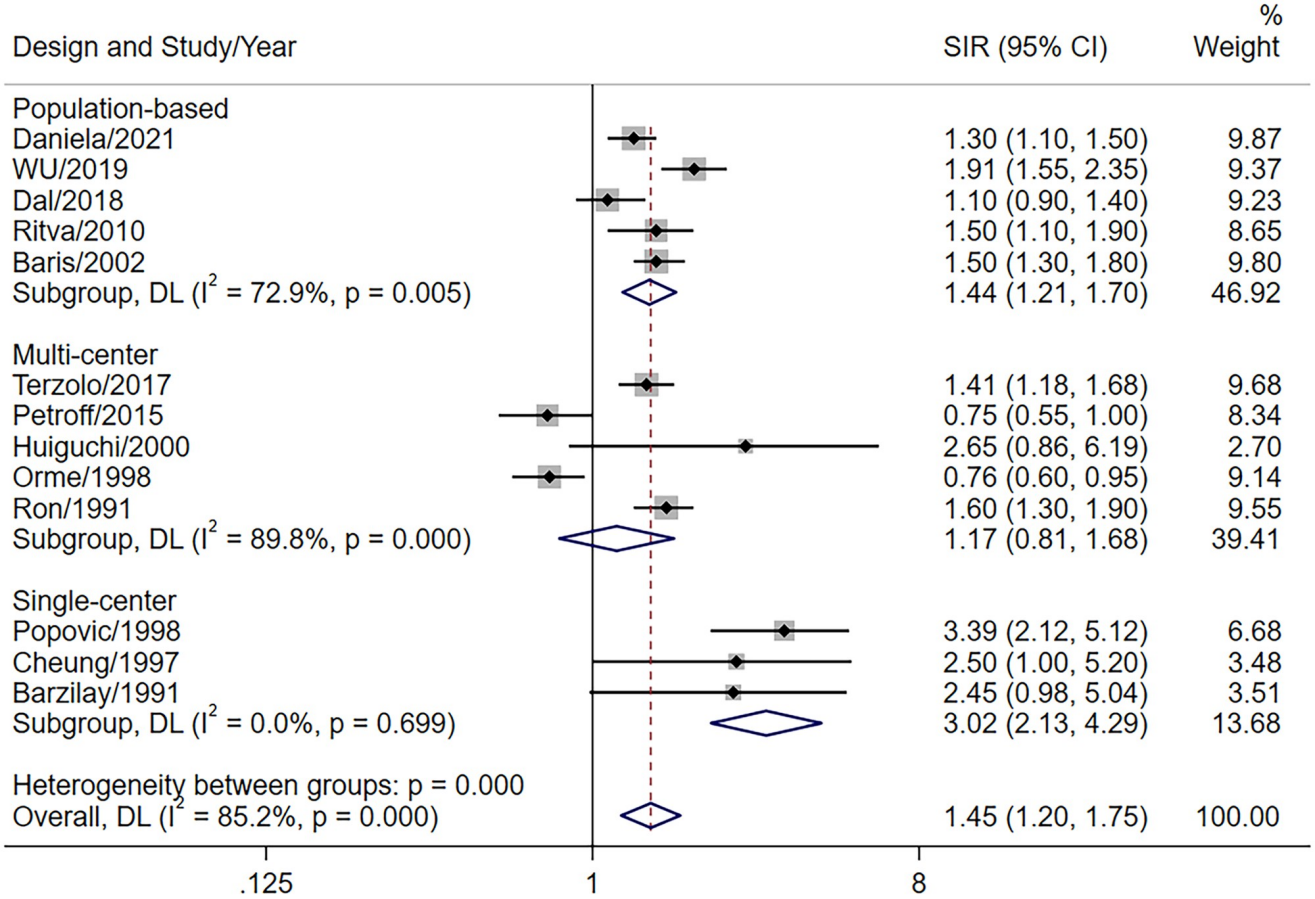
Pooled SIR of overall cancer in patients with acromegaly.

Owing to the high heterogeneity between studies, the pooled SIR of 13 studies was used to report the overall cancer risk using a random-effects model. We found a slightly increased risk of overall cancer in patients with acromegaly (SIR = 1.45, 95%CI = 1.20–1.75) as compared to the general population in all three study types: population-based (SIR = 1.44, 95%CI = 1.21–1.70); multi-center (SIR = 1.17, 95%CI = 0.81–1.68); and single-center (SIR = 3.02, 95%CI = 2.13–4.29) (Fig 2). With the high correlation found in the single-center studies in previous studies and our study, the risk of cancer was reduced after the deletion of the six single-center studies, although the conclusion remained the same (S2 Table).

Two of the most controversial cancers had varying degrees of increased risks. In the random-effects model, thyroid cancer risk in acromegaly patients was 6.96 times higher than that of the general population (SIR = 6.96, 95%CI = 2.51–19.33), whereas the risk of colorectal and anal cancer was nearly double (SIR = 1.95, 95%CI = 1.32–2.87). Notably, we demonstrated an association between acromegaly and the development of brain and central nervous system tumors (SIR = 6.14, 95%CI = 2.73–13.84), mainly meningioma, malignant astrocytoma, and schwannoma. Acromegaly was also associated with a high risk of urinary cancer (SIR = 2.66, 95%CI = 1.88–3.76) and hematological cancer (SIR = 1.89, 95%CI = 1.17–3.06) using the random effects model. The urinary system included kidney, ureter, and bladder cancer, and the hematological system mainly included malignant lymphomas, leukemia, and multiple myeloma. In gastrointestinal cancers other than colorectal and anal cancer, we revealed that acromegaly was associated with gastric cancer (SIR = 3.09, 95%CI = 1.47–6.50), using a random-effects model. With low heterogeneity between studies, the risk of pancreatic and small intestine cancer was also shown to be increased in the fixed-effects model (SIR = 2.59, 95%CI = 1.58–4.24). However, no association between acromegaly and hepatobiliary cancer was found (SIR = 1.48, 95%CI = 0.83–2.64). Moreover, acromegaly was related to an increased risk of connective tissue cancer (SIR = 3.15, 95%CI = 1.18–8.36) using a fixed-effects model, with no heterogeneity found between studies.

**Fig 3:**
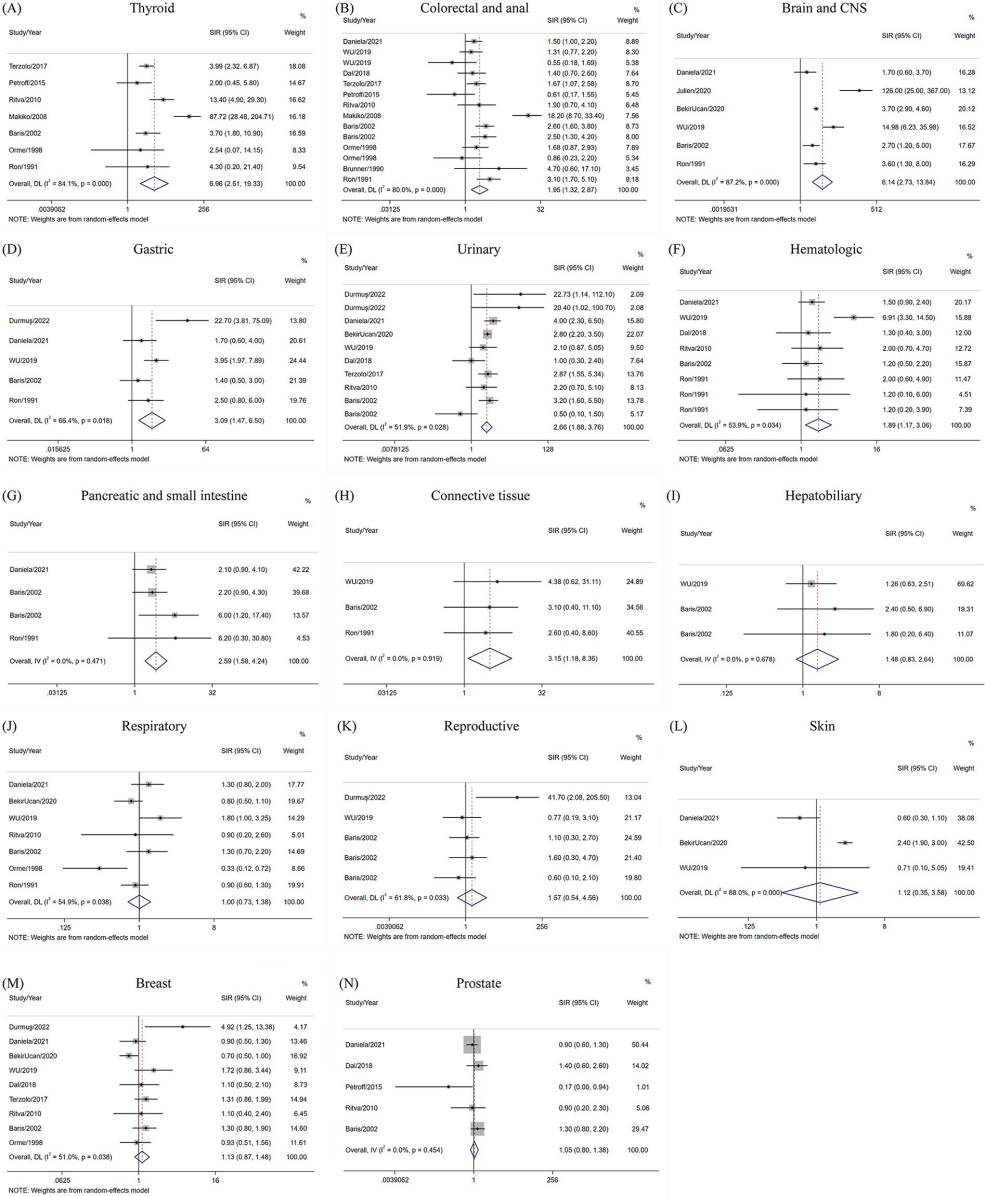
Pooled SIRs of various significant cancers in patients with acromegaly.

Seven studies analyzed acromegaly and respiratory system cancer within a random-effects model. The findings indicated that acromegaly was not related to respiratory cancer (SIR = 1.00, 95%CI = 0.73–1.38), mainly including lung cancer. No increased risk of reproductive system cancer (SIR = 1.57, 95%CI = 0.54–4.56), mainly including uterus, ovarian, and testicular cancers, was found. Acromegaly was also not associated with breast cancer (SIR = 1.13, 95%CI = 0.87–1.48), prostate cancer (SIR = 1.05, 95%CI = 0.80–1.38), or skin cancer (SIR = 1.12, 95%CI = 0.35–3.58). The fixed effects model was used for prostate cancer, and the random effects model was used for the remaining cancers (Fig 3).

**Fig 4:**
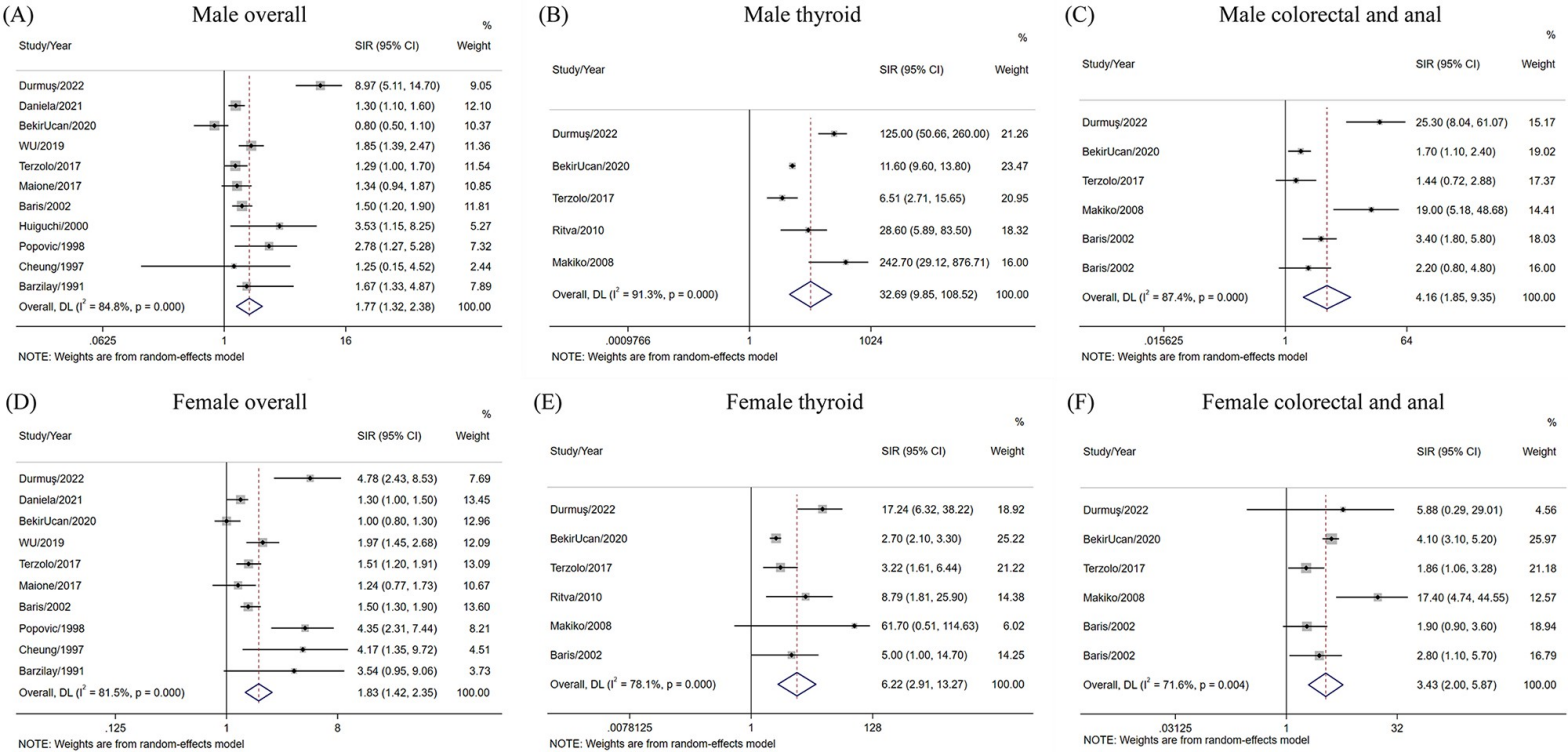
Pooled SIRs of cancer in patients with acromegaly according to sex.

As for the association between acromegaly and sex, the results successfully shed light on the association between acromegaly and enhanced risk of cancer in both sexes within a random effects model. For males, the overall incidence of cancer (SIR = 1.77, 95%CI = 1.32–2.38), as well as that of thyroid cancer (SIR = 32.69, 95%CI = 9.85–108.52), and colorectal and anal cancer (SIR = 4.16, 95%CI = 1.85–9.35), was higher among patients with acromegaly than among the general population. The same was true for females patients in terms of the overall incidence of cancer (SIR = 1.83, 95%CI = 1.42–2.35), as well as that of thyroid cancer (SIR = 6.22, 95%CI = 2.91–13.27), colorectal and anal cancer (SIR = 3.43, 95%CI = 2.00–5.87) (Fig 4).

### Publication bias

Egger’s test was used to estimate possible publication bias. The findings showed that, among females, except for the overall incidence of cancer (*P* = 0.021), thyroid cancer (*P* = 0.046), single-center nature (*P* = 0.031), and connective tissue cancer (*P* = 0.028), the *P*-values of Egger’s test were all higher than 0.05, suggesting that, in addition to the aforementioned four types of cancers, there was no notable publication bias in the results. As for the aforementioned four types of cancer, we used a trim-and-fill analysis to assess the effect of publication bias on the results. Publication bias for the overall incidence and incidence of connective tissue cancers was considered to have little impact on the pooled SIRs and 95%CI among females. Therefore, for females, the findings other than those for thyroid cancer, were stable (S3 Table).

### Sensitivity analysis

Sensitivity analysis results showed that Wu et al. [15] and Baris et al. [11] significantly impacted the pooled SIR in the analysis of connective tissue cancer. The SIRs and 95%CIs for other cancers were not significantly affected by any single study. Based on the above-mentioned results, meta-analysis results were generally stable (S1 Fig).

## Discussion

Our findings supported an increased cancer rate in acromegaly. The incidence of cancer was higher in single-center studies than in other types of studies. There was a modest reduction in cancer risk after we deleted the single-center studies, possibly because of the insufficient number of patients in some single-center studies and the fact that most patients in tertiary referral hospitals were possibly severely ill. Population-based studies are generally considered to have the highest quality and to reflect the cancer risk in patients with rare diseases best [28]. In our analysis, the pooled SIRs of population-based studies were similar to the overall SIR. Hence, our data indicate that the overall incidence of cancer was mildly elevated in acromegaly patients, which was consistent with the results of the study by Dal et al. [7].

We found that acromegaly patients had a higher risk of thyroid, colorectal and anal, gastric, and urinary system cancers than did the general population. Our study revealed that the incidence of thyroid cancer in acromegaly patients was 6.96 times that in the general population. However, in the research by Dal et al., the risk of thyroid cancer was increased 8.2 times among acromegaly patients as compared to the general population (SIR = 9.2, 95%CI = 4.2–19.9), which was significantly higher than our result. This difference may be due to the studies we included, as we only selected studies in which the expected number of SIRs were calculated using representative populations. The control groups of some studies were not representative, such as in the study conducted by Wolinski et al. [6] in 2017, where the control group included patients with hormonally inactive pituitary lesions, which may lead to an overestimation of the incidence of acromegaly.

Thyroid cancer showed the greatest elevation in risk in agromegaly patients. However, there may have been some surveillance bias. Acromegaly causes goiter in patients. In another meta-analysis [29], the pooled odds ratio (OR) for thyroid lesions was 3.1 times higher in patients with acromegaly than in the general population (95%CI = 1.8–5.5), suggesting that the former population had a higher rate of thyroid lesions. The clinical practice guidelines for Endocrinology Practice [30] recommend thyroid ultrasonography in acromegaly patients with palpable thyroid nodules, which may lead to a higher diagnosis of some silent thyroid cancers. However, the risk of thyroid cancer varies greatly, depending on location and diet. Both excessive iodine intake and iodine deficiency may contribute to the development of thyroid cancer [31]. The CIs are wide. Although the increased incidence of acromegalic thyroid cancer is not controversial, the specific risk needs to be evaluated comprehensively in the context of local dietary habits. Dal et al. reported a slight increase in the rate of breast cancer among patients with acromegaly as compared to that in the general population (SIR = 1.6, 95%CI = 1.1–2.3). We analyzed 9 studies and found no correlation between acromegaly and breast cancer. Half of these studies were published after 2018, and we speculate that the outcome bias may have been caused by the small sample size and insufficient statistical power.

Most importantly, we have recently demonstrated that acromegaly increases the incidence of hematological, brain and central nervous system, connective tissue, and pancreatic and small intestine cancers. This phenomenon can be explained through several biological mechanisms. Patients with acromegaly have chronically high levels of GH and IGF-1. The two RAS/RAF and PI3K/AKT pathways downstream of IGF-1 are the main pathways involved in the pathogenesis of meningioma [32], multiple myeloma [33], pancreatic cancer [34], and other malignant tumors. This axis can also promote tumor development by boosting epithelial–mesenchymal transition [35] and angiogenesis [36], interfering with endocrine metabolism [37], immunoregulation [38], and cross-linking with other pathways [39]. Thyroid cancer among patients with acromegaly has been discussed for decades, although brain and central nervous system cancer (SIR = 6.14, 95%CI = 2.73–13.84) requires more research. Three of the 6 studies on these cancers included benign brain tumors. When we excluded these three articles, the risk was reduced (SIR = 4.49, 95%CI = 1.17–11.76). Owing to the small number of cancer patients observed and expected, the CI was very wide, and it was difficult to quantify this risk accurately. Acromegaly, malignant meningioma, malignant astrocytoma, and malignant schwannoma require more attention. Julien et al. [23] showed that acromegaly with MEN-1 may have an enhanced association with meningiomas. However, a history of radiotherapy and somatostatin analog therapy are both important confounders that should be discussed further. Moreover, we found that patients with acromegaly appeared to have the same risk of hepatobiliary, respiratory, reproductive system, skin, and prostate cancer as the general population, which contradicts the findings of previous cell studies [39, 40].

However, it is worth noting that various studies have shown a dose-dependent relationship between the risk of malignancy and IGF-1 levels. Vargas et al. [41] found a direct impact in the middle of the duration of exposure to the GH–IGF-1 axis on the occurrence of thyroid cancer, which shows that the effective control of the disease is a factor that cannot be ignored. However, the data source for most studies was the National Center for Disease Registries, and it is difficult to determine the disease remission rates for these patients.

We found that sex had little effect on the overall cancer rate of patients with acromegaly. However, the hazard of thyroid cancer was much higher in males than in females, and that for colorectal and anal cancers was also increased to a certain extent. Torre et al. [42] found that smoking (OR = 21.335, 95%CI = 2.345–194.085) and high levels of glycated hemoglobin (OR = 5.58, 95%CI = 1.275–20.074) were risk factors for cancer in acromegaly cases. Most smokers are males, which may indirectly contribute to their increased risk of cancer; therefore, more research to determine whether males need more frequent cancer screening is warranted. Blood glucose levels are also a non-negligible factor. Albertelli et al. [43] demonstrated a significant negative correlation between colon tumors and metformin intake (OR = 0.22, 95%CI = 0.06–0.77), confirming the view of Torre et al. As acromegaly is a chronic disease, these results highlight the importance of long-term disease surveillance in these patients.

In terms of publication bias and sensitivity analysis, we found no significant publication bias except for the overall incidence of cancer, thyroid cancer incidence, single-center studies, and connective tissue cancer in females. Using the trim-and-fill analysis, we found that publication bias for the other three types of cancer, except female thyroid cancer, did not significantly impact the results. In the sensitivity analysis, we found that the studies of Wu et al. [15] and Baris et al. [11] greatly influenced the pooled SIR and 95%CI of connective tissue cancer. However, we showed that publication bias pertaining to patients with connective tissue cancer had little effect on outcomes. Therefore, the pooled SIRs and 95%CI of thyroid cancer in females requires further explanation.

The meta-analysis performed in our study had a rigorous design and the analysis results were objective and scientific. However, this study still had some limitations. First, due to the small observed and expected numbers, the CIs for thyroid, brain and central nervous system cancers, and connective tissue cancers were wide. It is difficult to determine whether the increased risk of these cancers is large or small. Further research is needed to determine whether the risk of these cancers increases substantially. Second, the results may be influenced by unpublished studies; positive results are more likely to be published, and unpublished negative consequences may impact the overall conclusion. Third, given the differences in the organization criteria of the cancer groups in the study, there may be a risk associated with exclusion, although the diagnosis of acromegaly is always delayed. Finally, due to an insufficient data volume, other factors, including age, disease duration, and blood glucose level, were not analyzed along with sex and study design. Therefore, larger epidemiological studies are needed to resolve this problem.

## Conclusion

Our study confirmed a modest increase in the incidence of multiple malignancies among patients with acromegaly. In addition to the screening for 2 cancers recommended by the Endocrine Society’s clinical practice guidelines, systematic cancer screening may be needed, although the benefits may be limited based on the current finding of a modestly increased risk over that in the general population. Future studies are needed to identify the cancer-related risk factors in patients with acromegaly, to ensure individualized cancer surveillance.

## Supporting information

S1 Fig Sensitivity analysis of pooled SIRs for various cancers. (TIF)

S1 Table Cohen’s kappa index for all items in the NOS scale. (DOCX)

S2 Table Pooled SIRs for various cancers except for single-center. (DOCX)

S3 Table Egger’s test values for pooled SIRs for various cancers. (DOCX)

S1 File INPLASY protocol of this study. (PDF)

S1 Checklist PRISMA 2020 checklist. (DOCX)
