# Supplementary figures and images for: Risk of cancer in acromegaly patients: An updated meta-analysis and systematic review
Source: PLoS One. 2023 Nov 30;18(11):e0285335. doi: 10.1371/journal.pone.0285335 (PMC10688666; doi:10.1371/journal.pone.0285335)

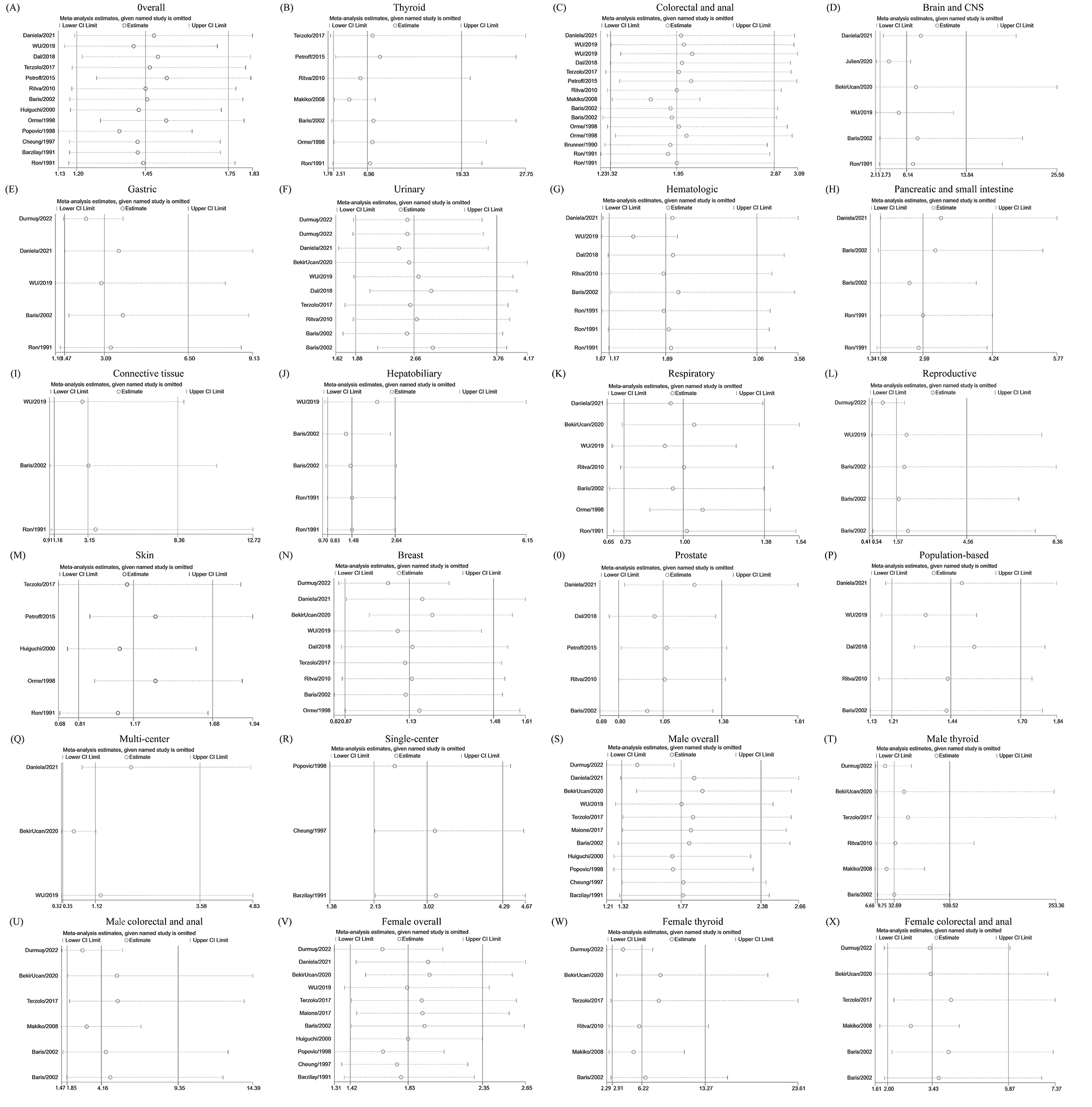

Supplement: S1 Fig — (TIF) [file pone.0285335.s001.tif]
