# Supplementary material for: Risk of cancer in acromegaly patients: An updated meta-analysis and systematic review
Source: PLoS One. 2023 Nov 30;18(11):e0285335. doi: 10.1371/journal.pone.0285335 (PMC10688666; doi:10.1371/journal.pone.0285335)
Supplement: S1 Table — (DOCX) [file pone.0285335.s002.docx]

**Supporting 1 Table. Cohen's kappa index for all items in the NOS scale**

|  | Cohen's kappa index | *P*-value |
| --- | --- | --- |
| **Selection** |  |  |
| Case definition adequate | 0.826 | <0.001 |
| Representativeness of the cases | 0.642 | <0.001 |
| Selection of Controls | 0.642 | <0.001 |
| Definition of Controls | 0.890 | <0.001 |
| **Comparability** |  |  |
| Comparability of cases and controls on the basis of the design or analysis | 0.683 | 0.003 |
| **Exposure** |  |  |
| Ascertainment of exposure | 1.000 | <0.001 |
| Same method of ascertainment for cases and controls | 1.000 | <0.001 |
| Non-Response rate | 1.000 | <0.001 |
