# Supplementary material for: Risk of cancer in acromegaly patients: An updated meta-analysis and systematic review
Source: PLoS One. 2023 Nov 30;18(11):e0285335. doi: 10.1371/journal.pone.0285335 (PMC10688666; doi:10.1371/journal.pone.0285335)
Supplement: S2 Table — (DOCX) [file pone.0285335.s003.docx]

**Supporting 2 Table. Pooled SIRs for various cancers except for single-center**

| Cancer | SIR (95% CIs) |
| --- | --- |
| Overall | 1.30(1.08-1.55) |
| Thyroid | 4.59(3.13-6.73) |
| Colorectal and anal | 1.70(1.34-2.16) |
| Brain and CNS | 3.86(2.24-6.66) |
| Gastric | 2.41(1.57-3.70) |
| Urinary | 2.51(1.85-3.42) |
| Hematologic | 1.89(1.17-3.06) |
| Pancreas and small intestine | 2.59(1.58-4.24) |
| Connective tissue | 3.15(1.18-8.36) |
| Hepatobiliary | 1.48(0.83-2.64) |
| Respiratory | 1.00(0.73-1.38) |
| Reproductive | 0.99(0.51-1.91) |
| Skin | 1.12(0.35-3.58) |
| Breast | 1.02(0.86-1.22) |
| Prostate | 1.05(0.80-1.38) |
