# Supplementary material for: Risk of cancer in acromegaly patients: An updated meta-analysis and systematic review
Source: PLoS One. 2023 Nov 30;18(11):e0285335. doi: 10.1371/journal.pone.0285335 (PMC10688666; doi:10.1371/journal.pone.0285335)
Supplement: S3 Table — (DOCX) [file pone.0285335.s004.docx]

**Supporting 3 Table. Egger's test values for pooled SIRs for various cancers**

| Cancer | Egger's test *P*-value |
| --- | --- |
| Overall | 0.431 |
| Female overall | **0.021** |
| Male overall | 0.218 |
| Thyroid | 0.899 |
| Female thyroid | **0.046** |
| Male thyroid | 0.240 |
| Colorectal and anal | 0.790 |
| Female colorectal and anal | 0.964 |
| Male colorectal and anal | 0.090 |
| Brain and CNS | 0.387 |
| Urinary | 0.941 |
| Hematologic | 0.888 |
| Gastric | 0.485 |
| Pancreas and small intestine | 0.126 |
| Connective tissue | **0.028** |
| Hepatobiliary | 0.383 |
| Respiratory | 0.787 |
| Reproductive system | 0.158 |
| Skin | 0.438 |
| Prostate | 0.563 |
| Breast | 0.056 |
| Population-based | 0.895 |
| Multi-center | 0.844 |
| Single-center | **0.031** |
