# Supplementary material for: Risk of cancer in acromegaly patients: An updated meta-analysis and systematic review
Source: PLoS One. 2023 Nov 30;18(11):e0285335. doi: 10.1371/journal.pone.0285335 (PMC10688666; doi:10.1371/journal.pone.0285335)
Supplement: S1 File — (PDF) [file pone.0285335.s005.pdf]

INPLASY PROTOCOL

To cite: Xiao et al. Risk of cancer in acromegaly patients: An updated meta-analysis and systematic review. Inplasy protocol 202340037. doi: 10.37766/inplasy2023.4.0037

Received: 12 April 2023

Published: 12 April 2023

Corresponding author:
Xiao ZheHao

734048795@qq.com

Author Affiliation:
The First Affiliated Hospital of Nanchang University.

Review Stage at time of this submission: **Completed but not published.**

Conflicts of interest:
None declared.

Risk of cancer in acromegaly patients: An updated meta-analysis and systematic review

Xiao, ZH[1]; Xiao, PP[2]; Wang, Y[3]; Fang, C[4]; Li, Y[5].

Review question / Objective: To analyze the risk of overall cancer and various types of cancer in patients with acromegaly compared to the general population.
Eligibility criteria: Inclusion criteria: 1. Studies that reported SIRs and 95% CIs. 2. Patients diagnosed with acromegaly. 3. The cancer incidence rate of representative populations calculated the expected number of SIRs. Exclusion criteria: 1. Studies that are not original articles, such as case reports and reviews. 2. Non-representative populations, including patients with specific diseases, used to calculate the expected number of SIRs.

INPLASY registration number: This protocol was registered with the International Platform of Registered Systematic Review and Meta-Analysis Protocols (INPLASY) on 12 April 2023 and was last updated on 12 April 2023 (registration number INPLASY202340037).

INTRODUCTION

Review question / Objective: To analyze the risk of overall cancer and various types of cancer in patients with acromegaly compared to the general population.

Rationale: To conduct a meta-analysis of the standardized incidence rates (SIRs) and 95% confidence intervals (CIs) of the overall and various significant cancers related to acromegaly to quantitatively evaluate the risk and tendency of cancer in patients with this disease.

Condition being studied: Acromegaly is a chronic endocrine disease, more than 90% caused by pituitary adenomas secreting growth hormones. Systemic multisystem disease of acromegaly lead to reduced life expectancy, and cancer has become the third cause of death in acromegaly, except for cardiovascular and respiratory diseases.

Xiao et al. Inplasy protocol 202340037. doi:10.37766/inplasy2023.4.0037 Downloaded from https://inplasy.com/inplasy-2023-4-0037/

METHODS

Search strategy: Using combined the following text words and MeSH terms as a search strategy: ("Neoplasm*" OR "Tumor*" OR "Neoplasia*" OR "Cancer*" OR "carcinoma*" OR "malignancy*" OR "tumor*") AND ("Acromegaly" OR ("Somatotropin Hypersecretion Syndrome*").

Participant or population: Patients diagnosed with acromegaly.

Intervention: Whether the patient has acromegaly.

Comparator: Representative populations, such as local population.

Study designs to be included: Observational study

Eligibility criteria: Inclusion criteria: 1. Studies that reported SIRs and 95% CIs. 2. Patients diagnosed with acromegaly. 3. The cancer incidence rate of representative populations calculated the expected number of SIRs. Exclusion criteria: 1. Studies that are not original articles, such as case reports and reviews. 2. Non-representative populations, including patients with specific diseases, used to calculate the expected number of SIRs.

Information sources: Relevant studies published from the time of database inception were obtained from the PubMed, Web of Science, and EMBASE databases.

Main outcome(s): The cancer standardized incidence ratios (SIRs) of acromegaly patients.

Additional outcome(s): None.

Data management: Endnote.

Quality assessment / Risk of bias analysis: Using the Newcastle-Ottawa scale checklist, and studies that scored ≥6 were considered to have high quality.

Strategy of data synthesis: The I2 test was used to assess heterogeneity. When heterogeneity was notable (P 50%), use a random-effects model. Otherwise, use a fixed-effects model.

Subgroup analysis: Cancer in patients with acromegaly compared to the general population divide in three study types (population-based; multi-center; and single-center) and gender (male; female) for subgroup analysis.

Sensitivity analysis: One article was excluded each time, and a meta-analysis pooled the remaining articles (n-1 articles) to assess the robustness and reliability of the pooled results. All tests were two-sided, and P-value <0.05 was regarded as statistically significant.

Language restriction: No.

Country(ies) involved: China (The First Affiliated Hospital of Nanchang University.

Keywords: Acromegaly; Cancer; Risk factor; Diabetes mellitus; Growth Hormone.

Contributions of each author:

Author 1 - Xiao Zhehao - Data collection, statistical analysis and original draft preparation.
Email: 734048795@qq.com
Author 2 - Xiao Pingping - Data collection, statistical analysis and original draft preparation.
Email: xpp7967@163.com
Author 3 - Wang Yong - Data collection, statistical analysis and original draft preparation.
Email: 1529845162@qq.com
Author 4 - Fang Cheng - Data collection, statistical analysis and original draft preparation.
Email: 2076252403@qq.com
Author 5 - Li Yong - Conceptualization and review and editing.
Email: liyongcsco@email.ncu.edu.cn

Support: National Natural Science Foundation of China (No.11664043), Natural Science Foundation of Jiangxi Province (No.20202BAB216031, No.20224AC

Xiao et al. Inplasy protocol 202340037. doi:10.37766/inplasy2023.4.0037 Downloaded from https://inplasy.com/inplasy-2023-4-0037/

B206026), The Traditional Chinese Medicine Administration Project of Jiangxi Province (No. 2022A140)..

Xiao et al. Inplasy protocol 202340037. doi:10.37766/inplasy2023.4.0037 Downloaded from https://inplasy.com/inplasy-2023-4-0037/
